# A Gleason score-related outcome model for human prostate cancer: a comprehensive study based on weighted gene co-expression network analysis

**DOI:** 10.1186/s12935-020-01230-x

**Published:** 2020-05-11

**Authors:** Yongzhi Wang, Zhonghua Yang

**Affiliations:** Department of Urology, Zhongnan Hospital of Wuhan University, Wuhan, 430071 China

**Keywords:** Prostate cancer, WGCNA, LASSO, Prognosis, TCGA, GEO

## Abstract

**Background:**

Prostate cancer (PCa) is the second leading cause of cancer death in men in 2018. Thus, the evaluation of prognosis is crucial for clinical treatment decision of human PCa patients. We aim to establishing an effective and reliable model to predict the outcome of PCa patients.

**Methods:**

We first identified differentially expressed genes between prostate cancer and normal prostate in TCGA-PRAD and then performed WGCNA to initially identify the candidate Gleason score related genes. Then, the candidate genes were applied to construct a LASSO Cox regression analysis model. Numerous independent validation cohorts, time-dependent receiver operating characteristic (ROC), univariate cox regression analysis, nomogram were used to test the effectiveness, accuracy and clinical utility of the prognostic model. Furthermore, functional analysis and immune cells infiltration were performed.

**Results:**

Gleason score-related differentially expressed candidates were identified and used to build up the outcome model in TCGA-PRAD cohort and was validated in MSKCC cohort. We found the 3-gene outcome model (CDC45, ESPL1 and RAD54L) had good performance in predicting recurrence free survival, metastasis free survival and overall survival of PCa patients. Time-dependent ROC and nomogram indicated an ideal predictive accuracy and clinical utility of the outcome model. Moreover, outcome model was enriched in 28 pathways by GSVA and GSEA. In addition, the risk score was positively correlated with memory B cells, native CD4 T cells, activated CD4 memory T cells and eosinophil, and negatively correlated with plasma cells, resting CD4 memory T cells, resting mast cells and neutrophil.

**Conclusions:**

In summary, our outcome model proves to be an effective prognostic model for predicting the risk of prognosis in PCa.

## Background

Prostate cancer (PCa) is the second leading cause of cancer death in men in 2018 [1]. Due to metastasis, the 5-year relative survival rate of distant PCa is only 30% [2]. Despite decades of efforts in research, the standard treatment options and guidelines for PCa patients diagnosed with metastatic progression have remained unchanged [3, 4]. Clinically, the Gleason scoring system has been widely used for assessment of prognosis of PCa based on histology [5]. However, in patients with metastatic PCa, metastatic biopsies are rarely performed [6]. Although prostate specific antigen (PSA) screening contributes to decrease PCa metastases and mortality [7], over-diagnosis and over-treatment become a controversial issue [8, 9].

With the development of precision medicine (PM), individualized treatment based on cancer genomic data has been achievable [10]. In view of the multiple treatment options and inconsistent outcomes of PCa, reliable biomarkers might help to optimize clinical decisions [11, 12]. For instance, as for diagnosis, the combination of digital-rectal examination and PSA value provides the risk stratification in most patients but could not give more details for the following steps. Therefore, prostate health indexes (PHI), four-kallikrein panel (4K), and even combination of PHI and 4K have been introduced [13–15]. The prognosis of PCa is greatly variable because of its various features [16]. However, there is little research about the biomarkers of PCa prognosis. PTEN, as a suppressor of prostate cancer, might be a prognostic biomarker for PCa [17]. Several miRNA, such as miR-145, has been demonstrated to suppress the androgen receptor in PCa cells and correlate to PCa prognosis [18]. However, there is still a huge distance between clinical application and these biomarkers because of lacking enough validation. Therefore, establishing an effective prognostic model for PCa has significant clinical implications.

The recent development of high-throughput profiling, sequencing technology and bioinformatics has revolutionized cancer research in general. By utilizing the publically available datasets and advanced bioinformatics tools, we aim to systematically explore biomarkers for PCa prognosis. The weighted gene co-expression network analysis (WGCNA) is an R package for weighted correlation network analysis and can be used as a data exploratory tool or a gene screening (ranking) method to find clusters (modules) of highly correlated genes [19]. It has been widely used to find hub genes in various cancers [20–22]. The least absolute shrinkage and selection operator (LASSO) method was originally designed for regression analysis. Lately, it has been applied to many fields, including the construction of prognostic models for various cancers [23–26].

In this study, we firstly used WGCNA to identify hub genes associated with Gleason score. By using LASSO method, we then constructed a 3-mRNA signature and extensively tested for predicting patient disease free survival. Meanwhile, the outcome model was validated in various independent datasets. Next, we built a nomogram based on the 3-mRNA signature combined with other clinical factors. We have confirmed the clinical utility by decision curve analysis. Furthermore, to explore tumor associated immune cell infiltration and risk score derived from outcome model, we used CIBERSORT to calculate the immune cell infiltration abundance and performed combination survival analysis.

## Materials and methods

### Clinical samples and data acquisition

Gene expression profiles of prostate cancer and corresponding clinical information of patients were obtained from UCSC Xena database (http://xena.ucsc.edu/), Gene Expression Omnibus (GEO) database (http://www.ncbi.nlm.nih.gov/geo/) and cbioportal database (http://www.cbioportal.org/). Detailed information for the 8 independent datasets including MSKCC, GSE116918, GSE46602, GSE54460, GSE70768, GSE70769, GSE16560 and GSE53922 were listed in Additional file 1: Table S1.

### Differentially expressed genes (DEGs) screening

Based on the pan-cancer normalized TCGA-PRAD data (498 prostate cancer samples and 52 non-tumor samples) in UCSC Xena database, we applied “limma” package in R to screen differentially expressed genes. Here, we set|log_2_ (Fold change)| > 1 and false discovery rate (FDR) < 0.05 as the cutoff.

### Weighted gene co-expression network construction and progression related genes identification

For WGCNA analysis, we used the gene expression matrix after the DEGs screening. Using “WGCNA” R package, we first deleted the outliers in each dataset [19]. Then, proper soft-thresholding parameter β was chosen to ensure a scale-free network, and genes with similar expression pattern were clustered into the same module. We then combined the modules with different clinical features (overall survival (OS) time, overall survival status, disease free survival (DFS) time, disease free survival status, age, biochemical recurrence, clinical M stage, clinical T stage, total Gleason score, laterality, number of positive lymphonode, pathological T stage, pathological N stage, PSA value, radiation therapy and targeted molecular therapy), we could finally identify the key genes related to total Gleason score.

### Establishment of outcome signature with LASSO regression model

LASSO (least absolute shrinkage and selection operator) is a regression analysis method that performs both variable selection and regularization in order to enhance the prediction accuracy and interpretability of the statistical model. Here, using “glmnet” package in R, we applied the LASSO Cox regression analysis to build an optimal prognostic signature for PCa by using key genes related to total Gleason score [27]. Due to few dead patients in the overall survival cohort of TCGA-PRAD, we therefore chose the disease free survival of TCGA-PRAD (n = 436) as the training set to set up the outcome model. Firstly, we excluded the patients without complete disease free survival information, then, optimal values of the penalty parameter lambda were determined through 10-times cross-validations. The minimum mean cross-validated error of the best lambda value was screened out. The risk score of prognostic signature for each sample was calculated by the relative expression of each prognostic gene in the signature and its associated coefficient. The risk score of the outcome signature = $\sum_{i = 1}^{n}$ (coef_i_ × Expr_i_), where Expr_i_ is the relative expression of the gene in the signature for patient i, coef_i_ is the LASSO coefficient of the gene i.

### Estimation of outcome signature for patients’ prognosis

According to the optimal p value, patients from different datasets were divided into low risk group and high risk group separately. Then we used “survivalROC” R package to perform time-dependent receiver operating characteristic curve (ROC) analysis and calculate the area under curve (AUC) for 1-year, 3-year and 5-year disease free survival, recurrence free survival (RFS), overall survival and metastasis free survival (MFS) for further evaluating the prediction accuracy for our model [28].

### Gene set enrichment analysis (GSEA) and gene set variation analysis (GSVA)

According to the optimal separation, we divided the TCGA-PRAD samples into high risk and low risk groups. Here, to further identify the role of our model in tumor metastasis, we used “clusterProfiler” and “fgsea” packages in R to visualize the correlation. P value and padj both < 0.05 were set as the cutoff. For the metastasis phenotype identification, we chose prostate cancer metastasis related gene sets as the reference; for the functional analysis, we chose Hallmark gene sets as the reference. For the GSVA analysis, we used “GSVA” R package and set t value > 2 and FDR < 0.05 as the cutoff [29]. Then the common gene sets were identified via GSEA and GSVA analysis.

### Construction and assessment of the nomogram

Excluding all missing information would lead to not enough patient samples. Therefore, we only firstly perform univariate cox regression analysis to identify the proper terms to build the nomogram. The forest was used to show the p value, HR and 95% CI of each variable through “forestplot” package in R. The nomogram, calibration plots and decision curve were generated using “rms” package. Afterwards, the calibration curves and decision curve analysis (DCA) were united to see whether our established nomogram was suitable for clinical utility.

### Evaluation of infiltrating immune cells and immune checkpoint with risk score

To evaluate the infiltration levels of immune cells in the PCa samples, we used the CIBERSORT algorithm [30], which provided an estimation of the abundances of member cell types by using gene expression data. B cells naïve, B cells memory, Plasma cells, T cells CD8, T cells CD4 naïve, T cells CD4 memory resting, T cells CD4 memory activated, T cells follicular helper, T cells regulatory (Tregs), T cells gamma delta, NK cells resting, NK cells activated, Monocytes, Macrophages M0, Macrophages M1, Macrophages M2, Dendritic cells resting, Dendritic cells activated, Mast cells resting, Mast cells activated, Eosinophils and Neutrophils were investigated. Furthermore, the correlation between risk score and immune cell infiltration was calculated and combination survival analyses were performed based on risk score and significantly-correlated infiltrating immune cells. Besides, the correlation between risk score and immune checkpoints were also investigated.

### Statistical analysis

R software 3.5.0 was used for all statistical analyses. Package details were listed in Additional file 2: Table S2. Statistical significance was set at probability values of p < 0.05. Two-tailed Student’s t-test was used for significance of differences between subgroups. One-way Anova test or Student t test were applied to analyze the correlation between risk score and clinicopathological parameters. Kaplan-Meier survival curves were built to analyze survival differences between the high risk group and low risk group. The ROC, calibration curve and DCA were compared for the predictive accuracy of the prognostic models.

## Results

### Differentially expressed genes (DEGs) screening and selection of candidate genes via WGCNA analysis

**Fig. 1:**
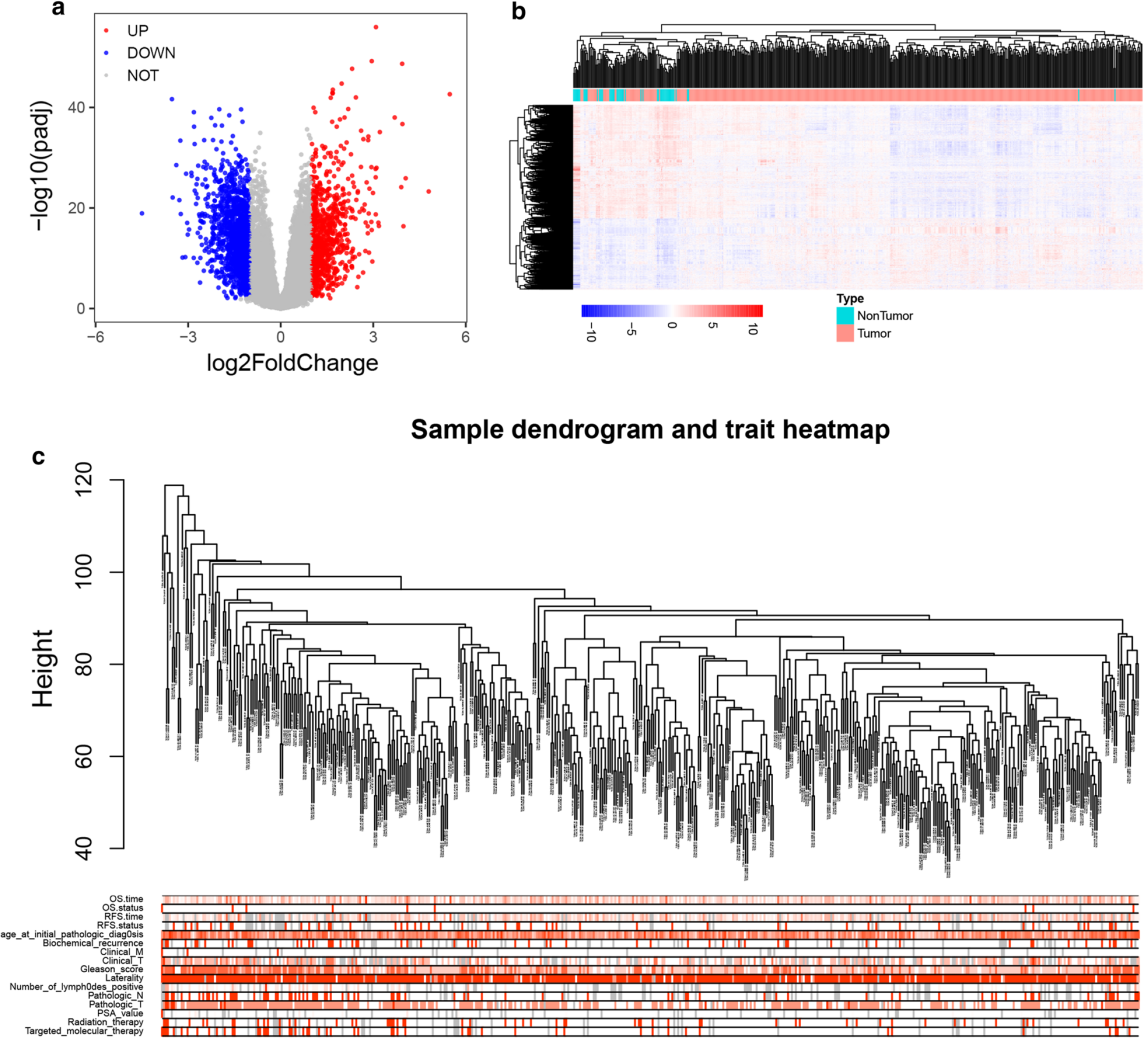
Identification of differentially expressed genes between prostate cancer and normal prostate in TGCA-PRAD and sample cluster of DEGs. **a** Volcano plot of the DEGs in TCGA-PRAD. Red dots represent up-regulated genes, blue dots represent down-regulated genes and grey dots represent genes with no significance. **b** Heatmap of DEGs in TCGA-PRAD. **c** Sample cluster of DEGs via average clustering method

**Fig. 2:**
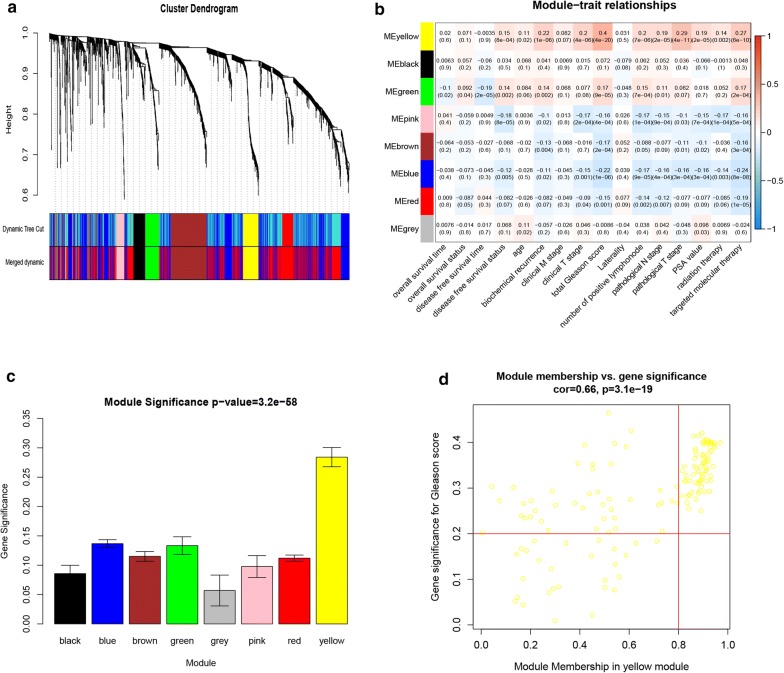
Identification of Gleason score-related candidate genes. **a** Dendrogram of all differentially expressed genes clustered. **b** Distribution of average gene significance in the modules. **c** The correlation between module eigengenes tumor grade. **d** Dotplot to screen hub genes in hub module. Genes in upper-right corner are hub genes associated with Gleason score

We identified 2245 DEGs (858 up-regulated and 1387 down-regulated genes) (Additional file 3: Table S3). Differentially expressed genes were visualized via volcano plot and heatmap (Fig. 1a, b) and further, we used DEGs to construct co-expression network. Here, based on the average clustering, we detected the outlier samples and no one was removed (Fig. 1c). Then, we chose β = 4 as the proper soft-thresholding parameter and built a scale-free network (Additional file 4: Figure S1). Combined with the module and clinical information, we eventually identified the yellow module which was highly correlated with total Gleason score (R = 0.4, p = 4E−20) and we subsequently selected the 73 hub genes in yellow module (Fig. 2 and Additional file 5: Table S4).

### Construction of outcome model

**Fig. 3:**
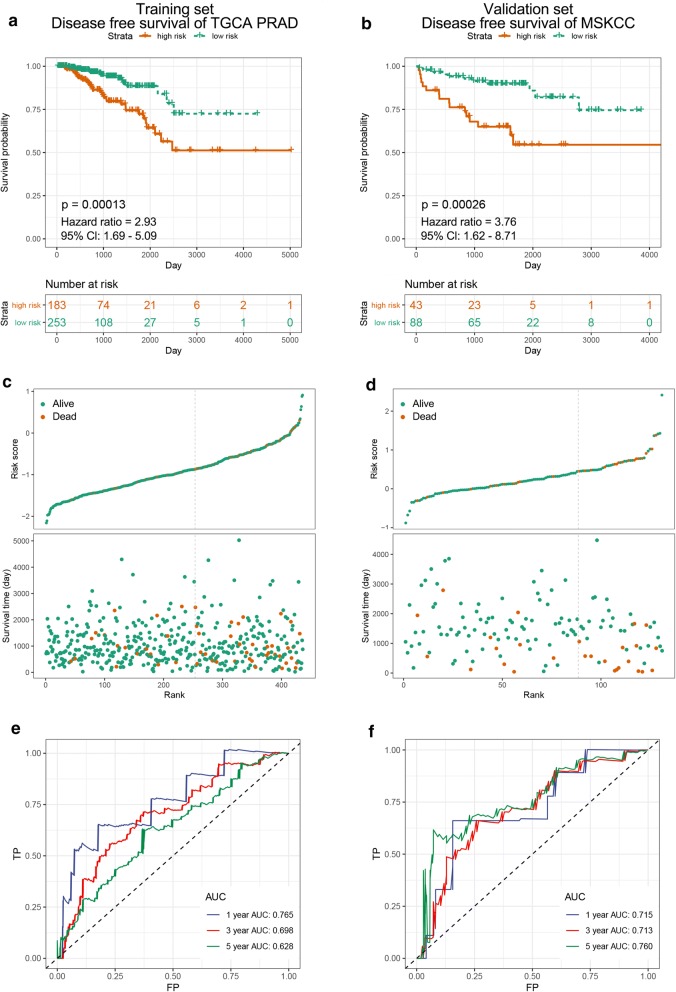
Risk score derived from 3-gene signature is a prognostic biomarker for disease free survival (DFS). **a**, **c**, **e** KM survival, risk score and time-dependent ROC curves of DFS in TCGA-PRAD training cohort. **b**, **d**, **f** KM survival, risk score and time-dependent ROC curves of DFS in MSKCC validation cohort. The high-risk and low-risk groups were stratified at optimal cut-off due to the risk score. The AUC was assessed at 1, 3 and 5 years

Excluding the samples with incomplete disease free survival data, we finally used 436 TCGA-PRAD patients as the training set. Using LASSO cox regression, we then generated a formula based on the expression of the three genes to predict DFS in TCGA-PRAD training dataset, and the formula for the risk score in each sample was calculated as follows: risk score = 1.562E−01*Exp_CDC45_ + 2.850E−02* Exp_ESPL1_ + 1.148E−04* Exp _RAD54L_ (Additional file 6: Figure S2). Meanwhile, expression levels of these 3 genes were shown in Additional file 7: Figure S3. According to the risk score, patients were stratified into low-risk and high-risk groups at the best separation cut-off. KM curves indicated that high-risk groups were significantly associated with poorer DFS and low-risk groups were associated with better DFS (p = 0.00013) (Fig. 3a, c). Time-dependent ROC analysis showed that the area under the ROC curve (AUC) for DFS in TCGA-PRAD cohort was 0.765 at 1 year, 0.698 at 3 years and 0.628 at 5 years (Fig. 3e).

### Validation of prognostic model

**Fig. 4:**
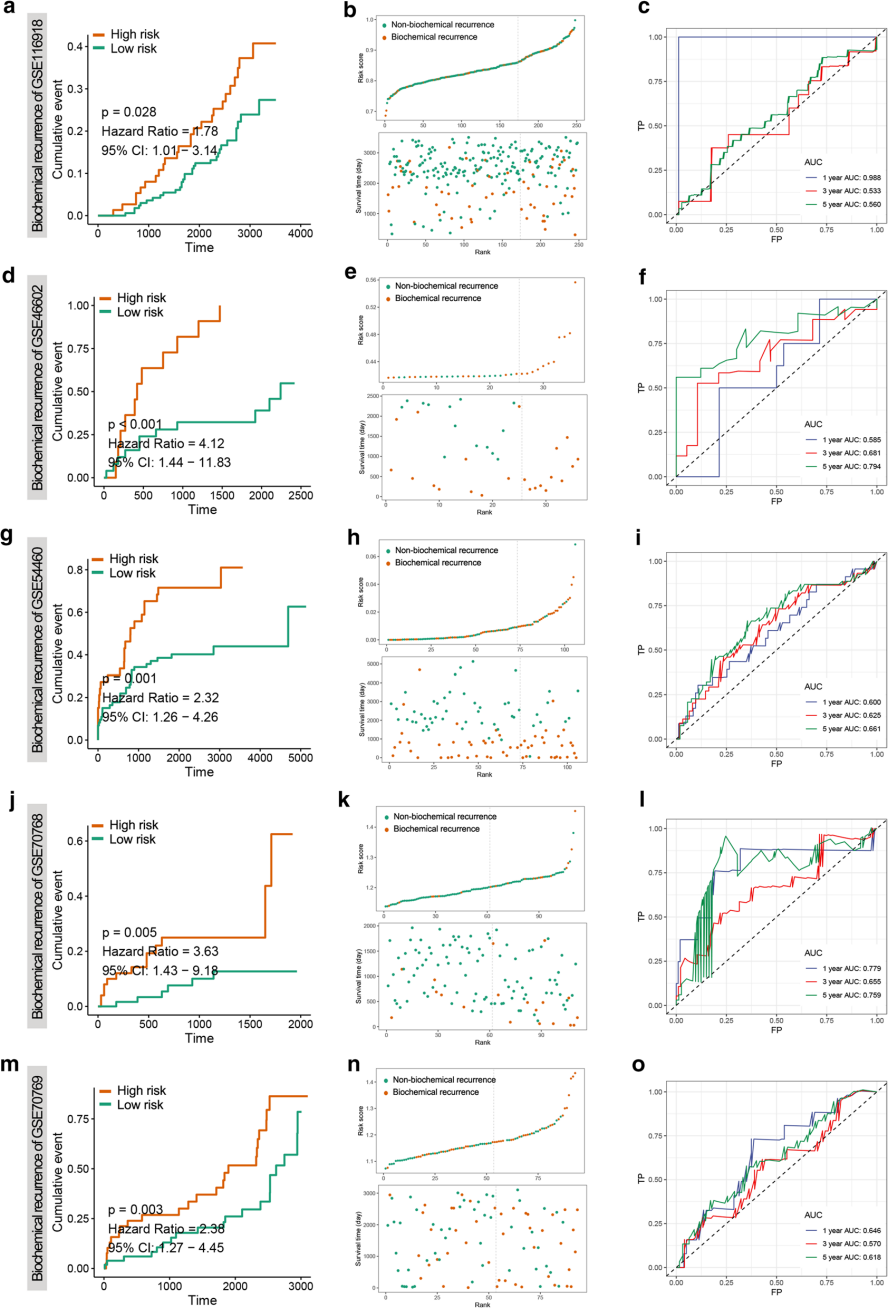
Risk score derived from 3-gene signature is a prognostic biomarker for recurrence free survival (RFS). **a**–**c** KM survival, risk score and time-dependent ROC curves of RFS in GSE116918 validation cohort. **d**–**f** KM survival, risk score and time-dependent ROC curves of RFS in GSE46602 validation cohort. **g**–**i** KM survival, risk score and time-dependent ROC curves of RFS in GSE54460 validation cohort. **j**–**l** KM survival, risk score and time-dependent ROC curves of RFS in GSE70768 validation cohort. **m**–**o** KM survival, risk score and time-dependent ROC curves of RFS in GSE70769 validation cohort. The high-risk and low-risk groups were stratified at optimal cut-off due to the risk score. The AUC was assessed at 1, 3 and 5 years

To validate our outcome model, MSKCC cohort was used for the DFS validation. We could observe the same significant prognostic value with p = 0.00026 and AUC with 1-, 3- and 5-year prognostic accuracies were 0.715, 0.713, 0.760, respectively (Fig. 3b, d, e). Since the total Gleason score was correlated with tumour behaviour, we then investigated our model’s role in overall survival and recurrence free survival. Due to few dead patients in TCGA-PRAD OS cohort, we didn’t include this cohort and used 2 independent cohorts to prove its utility in OS prediction. The results from the two OS validation datasets (GSE16560 and GSE53922) showed significant prognostic values were p = 0.005 and p = 0.032, respectively. The AUC of each dataset was 0.606 and 0.585 at 1 year; 0.562 and 0.552 at 3 years; 0.608 and 0.495 at 5 years, respectively (Additional file 8: Figure S4). Moreover, in the 5 validation cohorts (GSE116918, GSE46602, GSE54460, GSE70768 and GSE70769) for RFS, we could obviously see the significant outcomes that all the high-risk groups were associated with the poorer prognosis (Fig. 4a, d, g, j, m). The significant prognostic values of 5 RFS validation cohorts were p = 0.028, p < 0.0001, p = 0.001, p = 0.005 and p = 0.003, respectively. The AUC of each dataset was 0.988, 0.585, 0.600, 0.779 and 0.646 at 1 year; 0.533, 0.681, 0.625, 0.655 and 0.570 at 3 years; 0.560, 0.794, 0.661, 0.759 and 0.618 at 5 years, respectively (Fig. 4).

### Metastasis phenotype identification and metastasis free survival validation

**Fig. 5:**
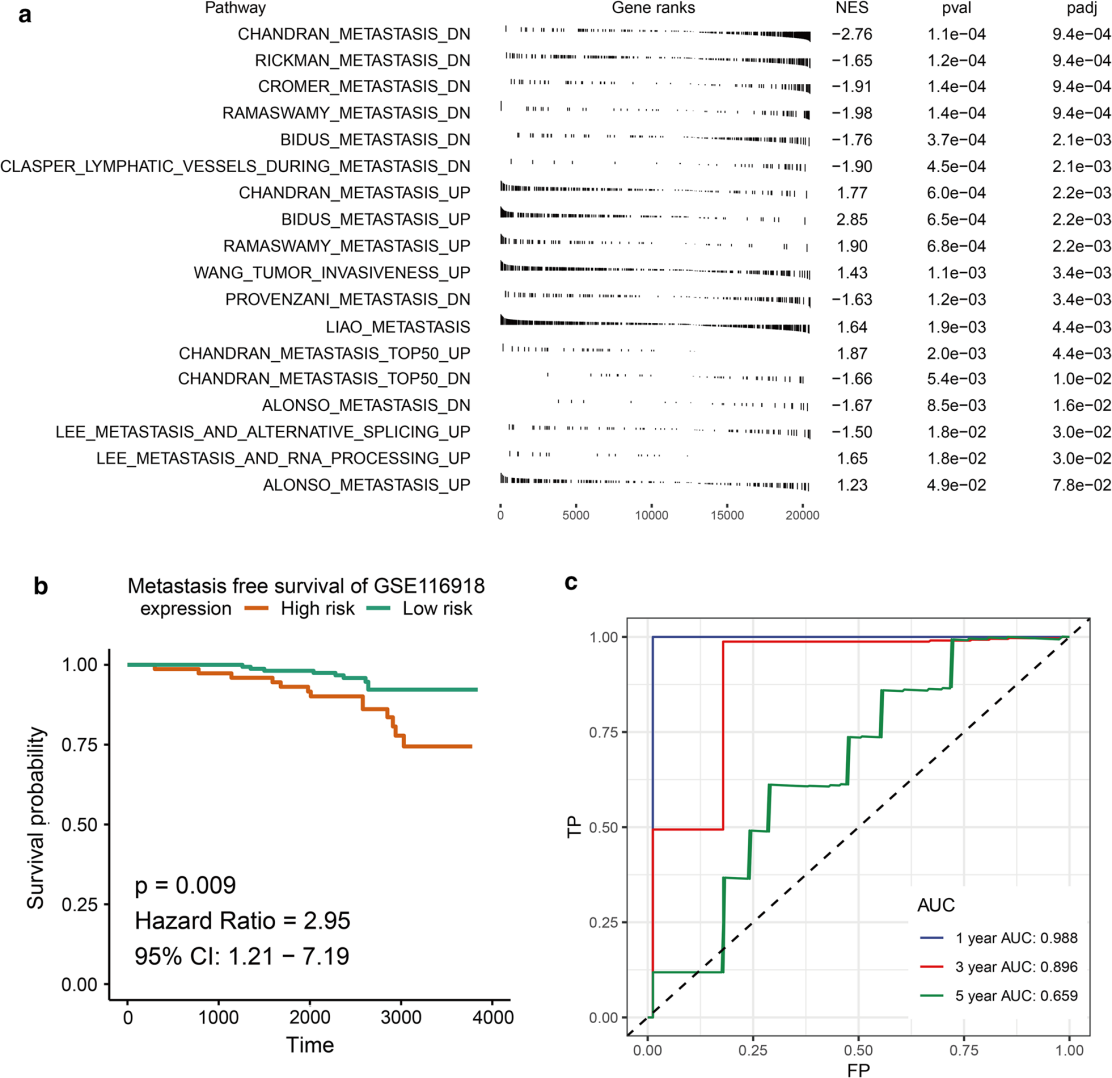
Risk score derived from 3-gene signature reveals a metastatic phenotype and is a prognostic biomarker for metastasis free survival (MFS). **a** Fgsea plot reveal the correlation between risk score and metastasis phenotype in prostate cancer. **b, c** KM survival and time-dependent ROC curves of MFS in GSE116918 validation cohort

Since Gleason score was correlated with tumor invasion, thus we used GSEA analysis to evaluate the correlation between metastasis and our model. We could find that high risk score was positively correlated with metastasis up and negatively correlated with metastasis down gene sets (Fig. 5a). Then the MFS validation set GSE116918 revealed that high-risk groups were significantly associated with poorer MFS and low-risk groups were associated with better MFS (p = 0.009) (Fig. 5b). Time-dependent ROC analysis showed that the area under the ROC curve (AUC) for MFS in GSE116918 cohort was 0.988 at 1 year, 0.896 at 3 years and 0.659 at 5 years (Fig. 5c).

### Correlation between the risk score with other clinicopathological characteristics

Clinicopathological data, including age, clinical M stage, clinical T stage, total Gleason score, laterality, number of positive lymphonodes, pathological N stage, pathological T stage, PSA value, radiation therapy and targeted molecular therapy were collected from TCGA-PRAD dataset. The detailed information of patients’ clinicopathological **characteristics** in TCGA-PRAD cohort were displayed in the Additional file 9: Table S5. Comparison results between risk score and different clinicopathological characters were shown in Additional file 10: Figure S5. In terms of clinical features, risk score was robustly increased in patients with lymphovascular invasion, more advanced stage and Gleason score and additional therapy, which indicated risk score was significantly positive correlated with tumour malignancy.

### Subgroup analysis of prognostic value of the outcome model

To check the good applicability of our outcome model, the stratification survival analyses were performed. The prognosis of the high-risk group in different age, early clinical stage, late pathological stage, pathological N- stage and different lymphnodes positive subgroups were still worse than the low-risk group (Additional file 11: Figure S6a, b, e, h, i, k, l). Although there was no significance between different Gleason score groups, late clinical stage, early pathological stage and pathological N + stage, we could still either find a worse prognostic trend in high risk group or more high-risk patients classified into high risk level group (Additional file 11: Figure S6c, d, f, g, j).

### Univariate cox regression analyses for the model prognostic ability and nomogram construction

**Fig. 6:**
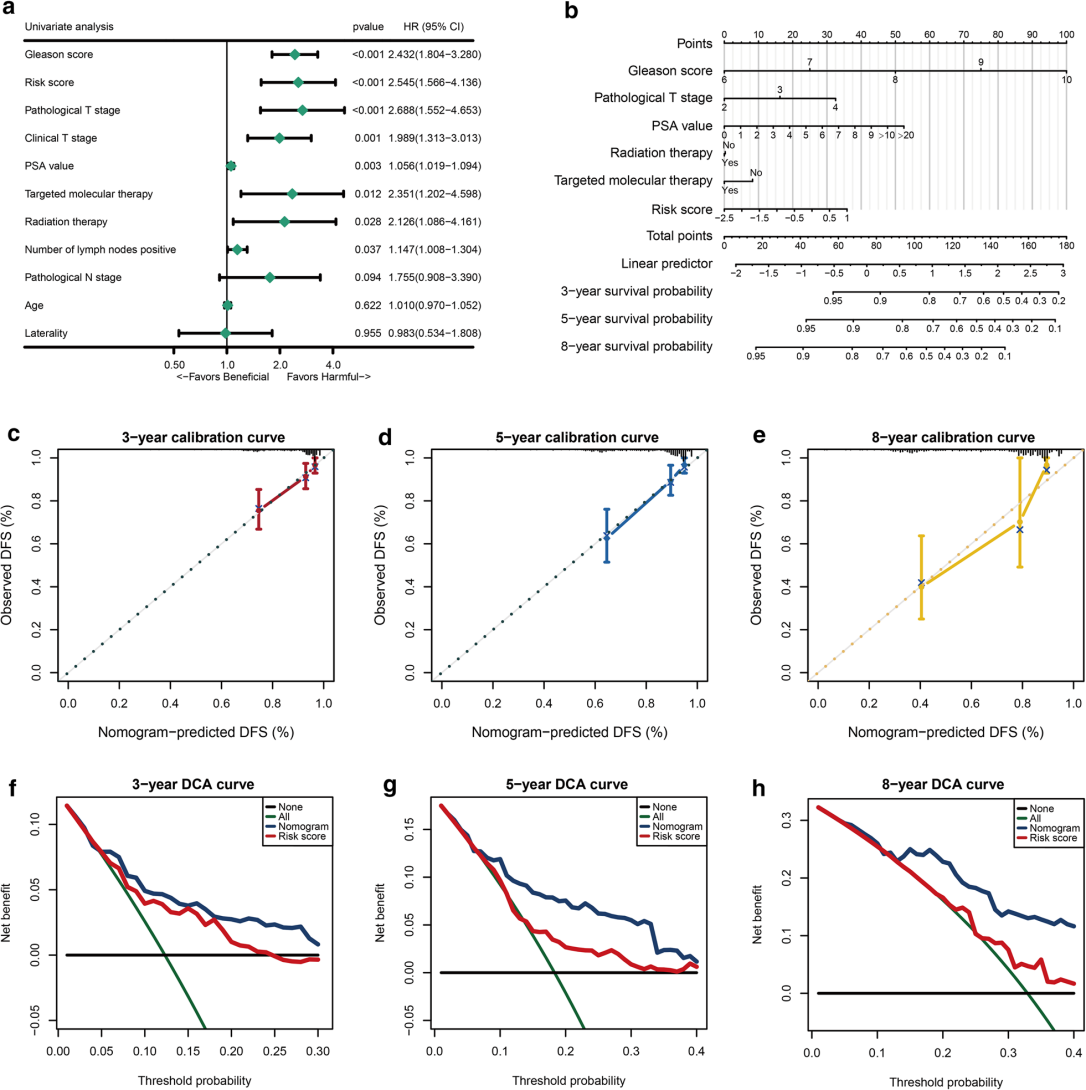
Risk score derived from 3-gene signature is an independent prognosis factor in the nomogram. **a** Forest plot summary of the univariate Cox analysis of risk score and clinicopathological characteristics. The blue diamond squares on the transverse lines represent the HR, and the black transverse lines represent the 95% CI. The p value and 95% CI for each clinical feature are displayed in detail. **b** Nomograms for predicting the probability of patient mortality at 3-, 5- or 8- year DFS based on risk score. **c**–**e** Calibration curves of the nomogram for predicting the probability of DFS at 3-, 5- and 8-year. **f**–**h** Decision curve analyses (DCA) curve of the nomograms based on TMERS risk-score for 3-year 5- and 8-year

We performed univariate cox regression analysis to investigate whether our model was a clinically independent prognostic factor for PCa patients. And from the unicox regression analysis, Gleason score, risk score, pathological T stage, clinical T stage, PSA value, targeted molecular therapy, radiation therapy and number of lymph nodes positive were significant (Fig. 6a). When we merged all clinical features to perform the multivariate cox regression, we found it would lose almost half of our included patients. Thus we skipped this step and used all significant variables to construct the nomogram which could provide a quantitative method for the clinicians to predict the probability of 3-, 5- and 8-year DFS in PCa patients (Fig. 6b). Every patient would get a total point by plus the each prognostic parameters point, and the higher total points mean a worse outcome for that patient. Moreover, the calibration curve indicated that good performance in the estimation of 3-, 5- and 8- year DFS of the nomogram compared with the estimation of Kaplan–Meier (Fig. 6c–e). The results of DCA analysis also demonstrated that our nomogram was of high potential for clinical usefulness (Fig. 6f–h).

### Functional enrichment analysis

**Fig. 7:**
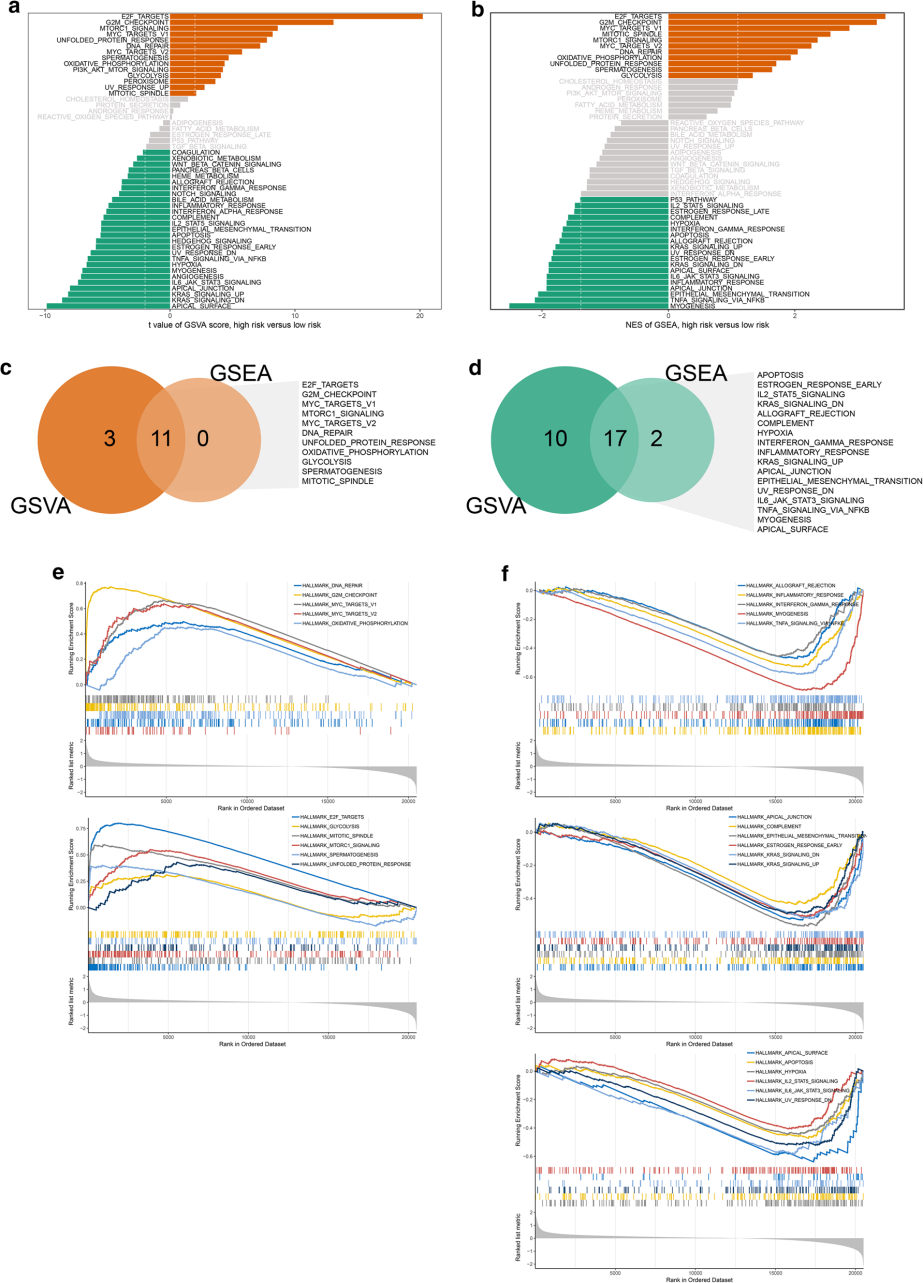
Functional analysis based on TCGA-PRAD. **a** Barplot of GSVA results. **b** Barplot of GSEA results. **c** Common activated gene sets. **d** Common suppressed gene sets. **e** GSEA plot of common activated gene sets. **f** GSEA plot of common suppressed gene sets

GSVA analysis showed that 30 gene sets were significantly changed, meanwhile 41 changed significantly via GSVA analysis (Fig. 7a, b and Additional file 12, 13: Tables S6, S7). We then chose the common activated or suppressed gene sets and eventually 11 activated gene sets (DNA repair, G2M checkpoint, MYC targets V1/2, oxidative phosphorylation, E2F targets, glycolysis, mitotic spindle, MTORC1 signaling, spermatogenesis and unfolded protein response) and 17 suppressed gene sets (allograft rejection, inflammatory response, interferon gamma response, myogenesis, TNFA signaling via NFKB, apical junction, complement, epithelial mesenchymal transition, estrogen response early, KRAS signaling UP/DN, apical surface, apoptosis, hypoxia, IL2/STAT5 signaling, IL6/JAK/STAT3 signaling and UV response DN) were identified (Fig. 7c–f).

### The landscape of immune infiltration in prostate cancer

**Fig. 8:**
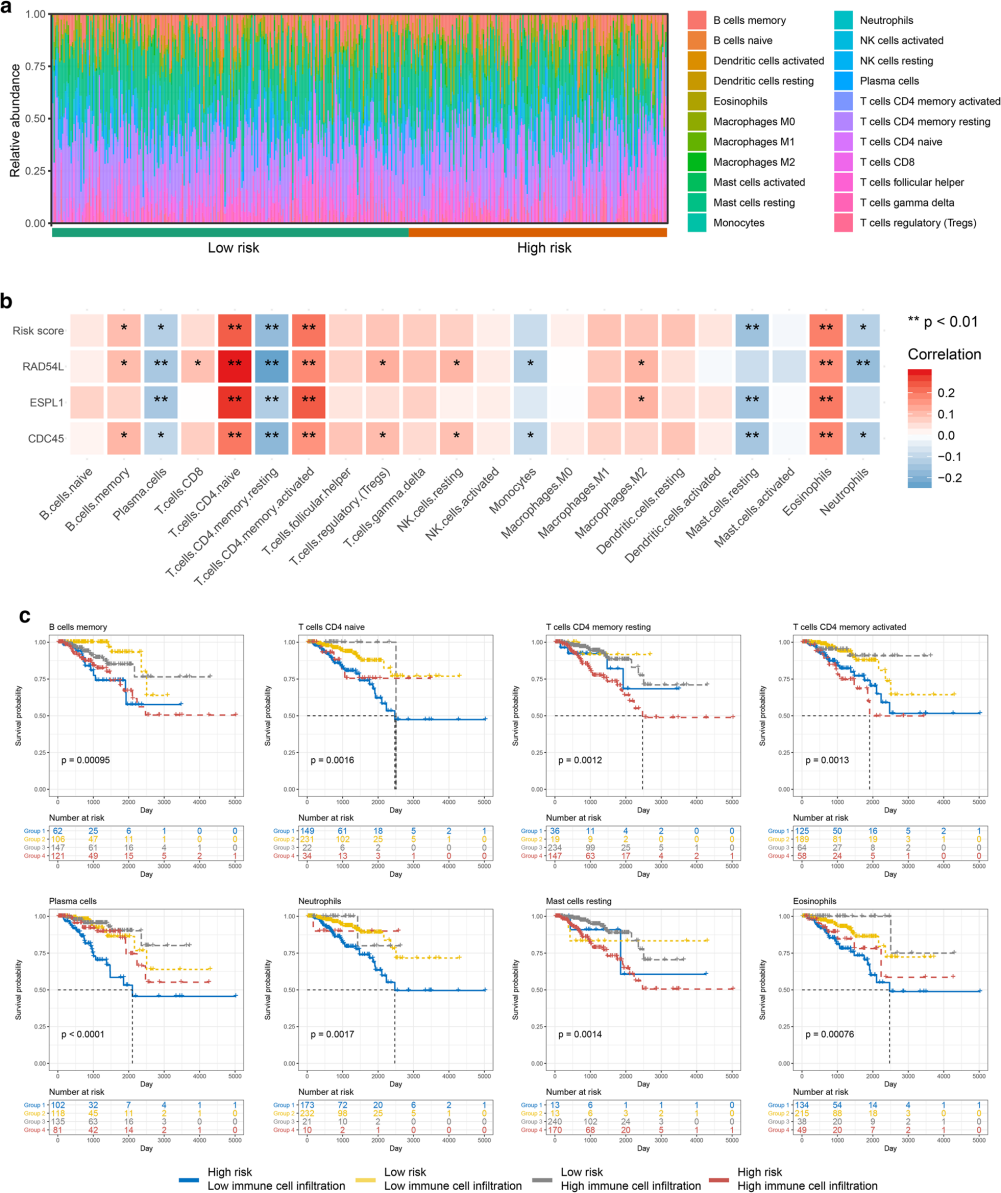
The landscape of immune infiltration and its correlation with risk score. **a** Relative abundance of immune cell infiltration in high- and low- risk groups. **b** Correlation matrix of risk score, Gleason score-related genes, and the amount of 22 types of immune cell. The blue indicated negative correlation, while red indicated positive correlation. Shading colour and asterisks represents the value of corresponding correlation coefficients. * p < 0.05, ** p < 0.01. **c** Combination KM survival with immune cell infiltration and risk score

Based on the CIBERSORT algorithm, we obtained an estimation of the abundances of 22 immune cells infiltrating in prostate cancer (Fig. 8a and Additional file 14: Table S8). We then calculated the correlation between immune cell infiltrations, risk score as well as 3 genes in the outcome model (Additional file 15: Table S9). The results showed that B cells memory, T cells CD4 native, T cells CD4 memory activated and eosinophil were positively correlated with our risk score and 3 genes in outcome model; while, plasma cells, T cells CD4 memory resting, mast cells resting and neutrophil were negatively correlated with our risk score and 3 genes in outcome model (Fig. 8b). Furthermore, we used those 8 immune cells to perform combination survival analysis with our risk score and we could find that each of them could divide patients into 4 groups which showed significant prognosis, especially plasma cell infiltration (Fig. 8c). As for the correlation between risk score and immune checkpoints, we found that risk score is significantly negative correlated with GAL9, LAG3, PD1LG2 and PDL1 (Additional file 16: Figure S7).

## Discussion

In recent years, quite a lot of promising biomarkers either for diagnosis or prognosis prediction were identified via high-throughput transcriptome profiling techniques. Although some gene signatures or nomograms have been established to predict the outcome for human prostate cancer, few of them used different independent validation cohorts [31–33].

In this study, we first identified differentially expressed genes between normal prostate and prostate cancer and then by using WGCNA analysis, we discovered a module highly-correlated with Gleason score and selected hub genes in this module. Based on the hub genes, we then constructed a 3-gene outcome model via LASSO cox regression analysis to predict the disease free survival of human PCa in TCGA-PRAD. Simultaneously, the patients were divided into high- and low-risk groups based on optimal cutoff and high risk group revealed a poor prognosis than low risk group, which could be validated in MSKCC cohort. We also successfully validated the effectiveness of model in predicting OS, RFS and MFS in 7 independent datasets. In addition, the nomogram based on the model exhibits an impressive performance and clinical applicability.

The genes (CDC45, ESPL1 and RAD54L) in our prognostic model have been previously reported to be associated with various cancers. However, only few papers revealed their roles in human prostate cancer. Li et al. reported that cell division cycle 45 (CDC45) might be useful markers for predicting tumor metastasis and therapeutic targets for the treatment of PCa patients via protein–protein network analysis [34]. Zhang et al. demonstrated that extra spindle pole bodies like 1 (ESPL1) could encode separase protein, which was up-regulated in numerous human cancers including breast, bone, brain, and prostate [35]. As for RAD54L, Li et al. found that castration-resistant prostate cancer (CRPC) cells showed a set of homologous recombination (HR) -associated genes, including BRCA1, RAD54L, and RMI2 were elevated [36].

Since our outcome model showed considerable power in risk stratification, the potential biological process and signaling pathways need to be investigated. By using GSEA and GSVA analysis, we identified several activated pathways which were highly correlated with cell cycle such as MYC targets, E2F targets, mitotic spindle and G2M checkpoint. Therefore, we supposed that outcome model derived cell cycle alteration might play a critical role in cancer progression which could lead to poorer prognosis in PCa patients. We also found some pathways associated with immune response and inflammation were suppressed which might suggest high-risk patients could have higher risk of immunosuppression.

The crosstalk of tumor and immune cells from the tumor microenvironment (TME) is essential for tumor progression and metastasis development [37, 38]. In this study, we also put emphasis on tumor immune cells infiltration. We calculated the correlation between immune cell abundance and risk score derived from outcome model. The 4 positive correlated immune cells were memory B cells, native CD4 T cells, activated CD4 memory T cells and eosinophil. It was reported that B-cells were activated, differentiate to a memory B-cell phenotype in high-grade serous ovarian cancer (HGSOC); they could be activated by DCs and promote a cytotoxic response and cause HGSOC metastases [39]. CD4^+^ T cells were reported to play a central role in initiating and maintaining anticancer immune responses in human head and neck cancer [40]; meanwhile, they were also reported to correlate with lymph node involvement and unfavorable prognosis in human breast cancers [41]. Therefore, we cannot just easily draw a conclusion as the immune cells infiltration was dynamic. The other 4 negative correlated immune cells were plasma cells, resting CD4 memory T cells, resting mast cells and neutrophil. Mast cells were immune cells that accumulated in the tumors and their microenvironment during disease progression. For instance, mast cells could secrete pro-angiogenic and growth factors but also pro- and anti-inflammatory mediators, thus it was really difficult to explain clearly its role in tumor progression [42]. As for the tumor-associated neutrophils (TAN), it functioned like a “double-edged sword” [43]. On one hand, it could acquire a tumor-promoting phenotype via induction by TGF-β [44]. On the other hand, it could mediate immunosuppression by inhibiting the tumor-killing function of cytotoxic T cells [45].

## Conclusions

In conclusion, our study was the first to construct an outcome model based on WGCNA analysis in human prostate cancer. Consisting of Gleason score related genes, our outcome model had promising potential in predicting prognosis of PCa. Meanwhile, some limitations of our study should be acknowledged. First, this is a retrospective bioinformatics analysis; large prospective clinical trials were needed to prove its real utility. Second, experimental studies are required to further explore underlying mechanisms of 3 candidate genes in our outcome model.

## Supplementary information

**Additional file 1: Table S1.** Information of all datasets in our study.

**Additional file 2: Table S2.** Detail information for packages.

**Additional file 3: Table S3.** Differentially expressed genes in TCGA-PRAD.

**Additional file 4: Figure S1.** Determination of soft-thresholding power in the weighted gene co-expression network analysis (WGCNA). (a) Analysis of the scale-free fit index for various soft-thresholding powers (β). (b) Analysis of the mean connectivity for various soft-thresholding powers. (c) Histogram of connectivity distribution when β = 4. (d) Checking the scale free topology when β = 4.

**Additional file 5: Table S4.** Hub genes correlated with Gleason score via WGCNA analysis.

**Additional file 6: Figure S2.** Construction of LASSO Cox regression model in TCGA-PRAD.

**Additional file 7: Figure S3.** Expression level of 3 Gleason score related genes. (a) Expression of 3 Gleason score related genes between prostate cancer and normal prostate in TCGA-PRAD. (b) Expression of 3 Gleason score related genes between different Gleason scores in TCGA-PRAD.

**Additional file 8: Figure S4.** Risk score derived from 3-gene signature is a prognostic biomarker for overall survival (OS). (a, c, e) KM survival, risk score and time-dependent ROC curves of OS in GSE16560 validation cohort. (b, d, f) KM survival, risk score and time-dependent ROC curves of OS in GSE53922 validation cohort. The high-risk and low-risk groups were stratified at optimal cut-off due to the risk score. The AUC was assessed at 1, 3 and 5 years.

**Additional file 9: Table S5.** Clinical information with DFS data of TCGA-PRAD.

**Additional file 10: Figure S5.** Association between risk score and clinicopathological characteristics. The survival rate of indicated subgroups in different clinicopathological characteristics was measured. Boxplots indicate the correlation between risk score and the indicated subtype of each clinicopathological characteristics by the t-test or one-way ANOVA. The patients are stratified into different subtypes based on (a) age: elder: age ≥ 65, younger: age < 65. (b) clinical M stage: cM0 and cM1. (c) clinical T stage: cT1, cT2, cT3 and cT4. (d) total Gleason score: 6, 7, 8, 9, 10. (E) laterality: left, right and bilateral. (f) number of positive lymph nodes by HE: number of positive lymph nodes by HE = 0 and number of positive lymphnodes by HE > 0. (g) pathological N stage: pN0 and pN1. (h) pathological N stage: pT2, pT3 and pT4. (i) PSA value: <10, 10-20 and >20. (j) Radiation therapy: yes and no. (k) targeted molecular therapy: yes and no.

**Additional file 11: Figure S6.** KM survival subgroup analyses of all patients in TCGA-PRAD cohort according to the risk score stratified by clinical characteristics. (a) Age < 65. (b) Age ≥ 65. (c) Gleason score 6-7. (d) Gleason score 8-10. (e) Clinical stage I-II. (f) Clinical stage III-IV. (g) Pathological stage I-II. (h) Pathological stage III-IV. (i) Pathological N stage - (N0). (j) Pathological N stage + (N+). (k) Number of positive lymph nodes by HE = 0. (l) Number of positive lymph nodes by HE > 0.

**Additional file 12: Table S6.** The results of GSEA analysis.

**Additional file 13: Table S7.** The results of GSVA analysis.

**Additional file 14: Table S8.** CIBERSORT Output matrix.

**Additional file 15: Table S9.** Correlation between risk score and immune cells infiltration.

**Additional file 16: Figure S7.** Correlation between risk score and immune checkpoints.

## Data Availability

All data generated or analysed during this study are included in this published article and Additional files.

## Abbreviations

PCa: Prostate cancer
DEGs: Differentially expressed genes
PSA: Prostate specific antigen
PHI: Prostate health indexes
4 K: Four-kallikrein panel
WGCNA: Weighted gene co-expression network analysis
GEO: Gene Expression Omnibus
FDR: False discovery rate
LASSO: Least absolute shrinkage and selection operator
ROC: Receiver operating characteristic curve
AUC: Area under curve
GSEA: Gene set enrichment analysis
GSVA: Gene set variation analysis
DCA: Decision curve analysis
Tregs: Regulatory T cells
OS: Overall survival
RFS: Recurrence free survival
MFS: Metastasis free survival
CDC45: Cell division cycle 45
ESPL1: Extra spindle pole bodies like 1
RAD54L: DNA repair and recombination protein RAD54-like
CRPC: Castration-resistant prostate cancer
HR: Homologous recombination
TME: Tumor microenvironment
HGSOC: High-grade serous ovarian cancer
TAN: Tumor-associated neutrophils
