# Supplementary material for: A Gleason score-related outcome model for human prostate cancer: a comprehensive study based on weighted gene co-expression network analysis
Source: Cancer Cell Int. 2020 May 11;20:159. doi: 10.1186/s12935-020-01230-x (PMC7216484; doi:10.1186/s12935-020-01230-x)
Supplement: Supplementary file 1 — Additional file 1: Table S1. Information of all datasets in our study. [file 12935_2020_1230_MOESM1_ESM.docx]

Supplementary table Information of all datasets included.

| GEO number | Platform | Sample number with survival data | Survival data |
| --- | --- | --- | --- |
| TCGA-PRAD | Illumina HiSeq 2000 | 436 | DFS |
| MSKCC | Cbioportal database | 131 | DFS |
| GSE116918 | Almac Diagnostics Prostate Disease Specific Array | 248 | BCR and MFS |
| GSE46602 | Affymetrix Human Genome U133 Plus 2.0 Array | 36 | BCR |
| GSE54460 | Illumina HiSeq 2000 | 106 | BCR |
| GSE70768 | Illumina HumanHT-12 V4.0 expression beadchip | 111 | BCR |
| GSE70769 | Illumina HumanHT-12 V4.0 expression beadchip | 92 | BCR |
| GSE16560 | Human 6k Transcriptionally Informative Gene Panel for DASL | 281 | OS |
| GSE53922 | Illumina HumanWG-6 v3.0 expression beadchip | 112 | OS |

­­­­
