# Supplementary material for: A Gleason score-related outcome model for human prostate cancer: a comprehensive study based on weighted gene co-expression network analysis
Source: Cancer Cell Int. 2020 May 11;20:159. doi: 10.1186/s12935-020-01230-x (PMC7216484; doi:10.1186/s12935-020-01230-x)
Supplement: Supplementary file 2 — Additional file 2: Table S2. Detail information for packages. [file 12935_2020_1230_MOESM2_ESM.docx]

|  | Version | web address |
| --- | --- | --- |
| limma | 3.42.2 | <https://bioconductor.org/packages/release/bioc/html/limma.html> |
| WGCNA | 1.66 | <http://horvath.genetics.ucla.edu/html/CoexpressionNetwork/Rpackages/WGCNA/> |
| glmnet | 2.0-16 | <http://www.jstatsoft.org/v33/i01/> |
| survivalROC | 1.0.3 | <https://cran.r-project.org/web/packages/survivalROC/index.html> |
| clusterProfiler | 3.14.3 | <https://bioconductor.org/packages/release/bioc/html/clusterProfiler.html> |
| fgsea | 1.12.0 | <https://bioconductor.org/packages/release/bioc/html/fgsea.html> |
| GSVA | 1.34.0 | <https://bioconductor.org/packages/release/bioc/html/GSVA.html> |
| forestplot | 1.7.2 | <https://cran.r-project.org/web/packages/forestplot/index.html> |
| rms | 5.1-2 | <http://biostat.mc.vanderbilt.edu/rms> |
