# Supplementary figures and images for: A Gleason score-related outcome model for human prostate cancer: a comprehensive study based on weighted gene co-expression network analysis
Source: Cancer Cell Int. 2020 May 11;20:159. doi: 10.1186/s12935-020-01230-x (PMC7216484; doi:10.1186/s12935-020-01230-x)

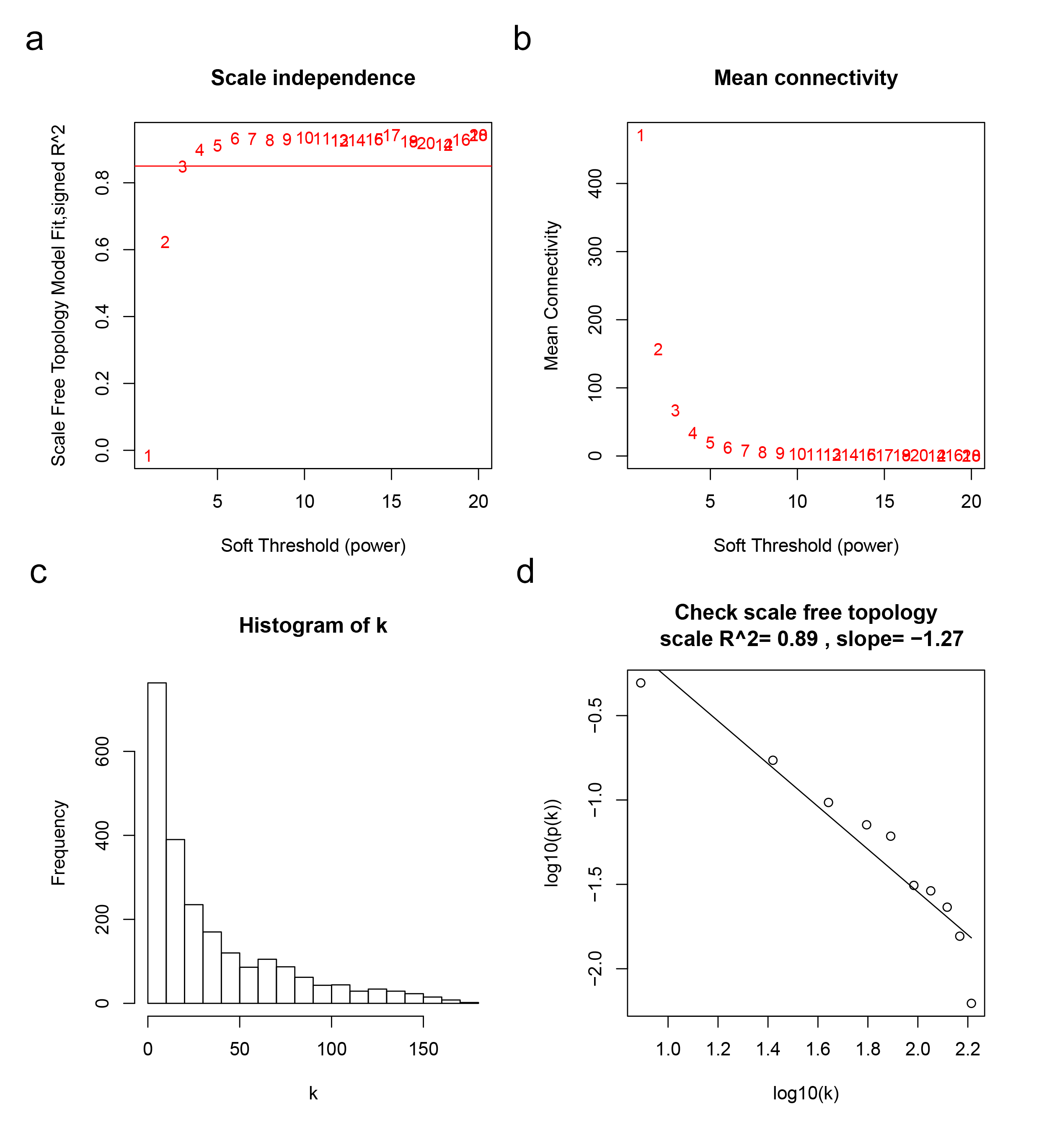

Supplement: Supplementary file 4 — Additional file 4: Figure S1. Determination of soft-thresholding power in the weighted gene co-expression network analysis (WGCNA). (a) Analysis of the scale-free fit index for various soft-thresholding powers (β). (b) Analysis of the mean connectivity for various soft-thresholding powers. (c) Histogram of connectivity distribution when β = 4. (d) Checking the scale free topology when β = 4. [file 12935_2020_1230_MOESM4_ESM.tif]

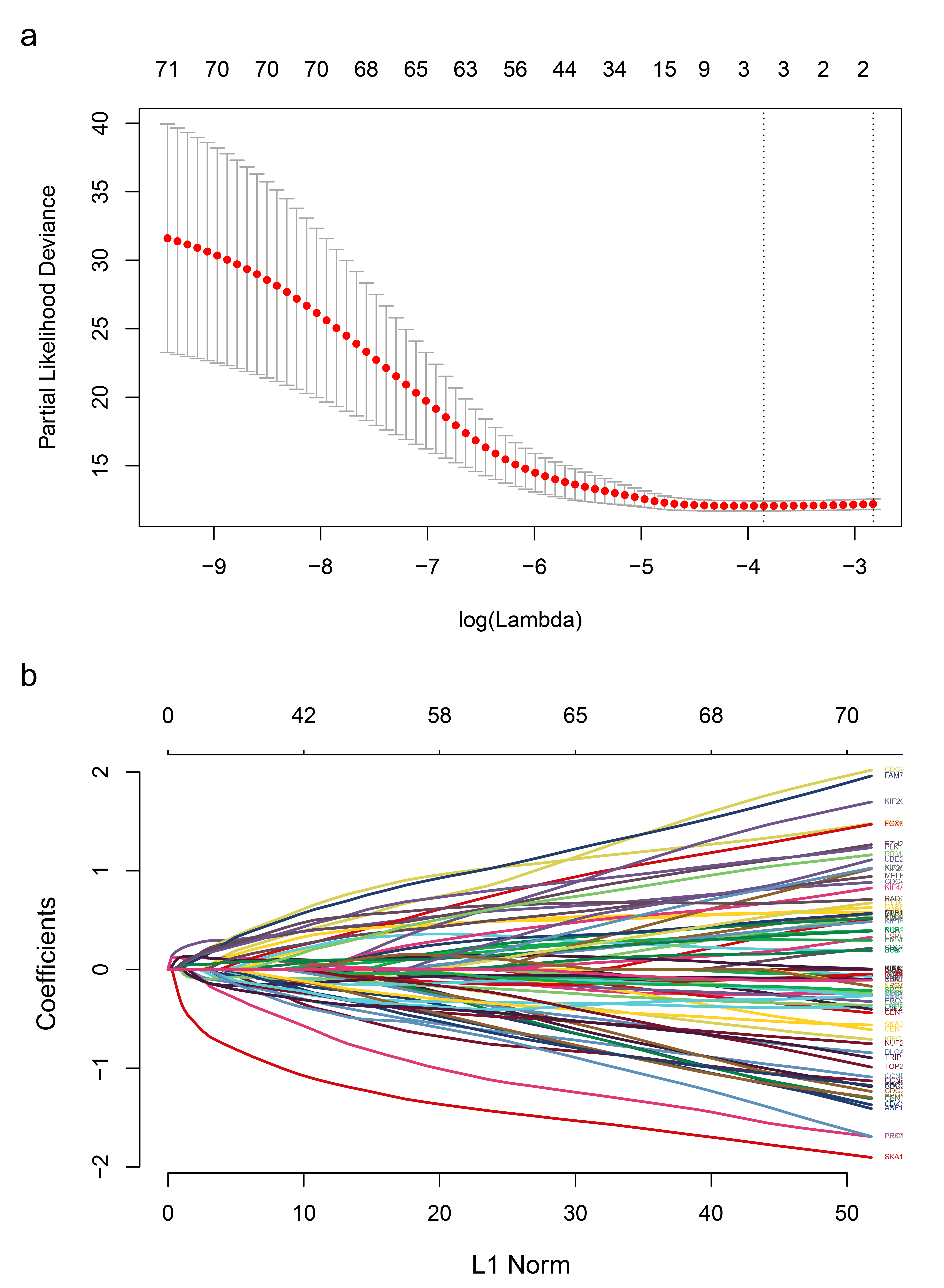

Supplement: Supplementary file 6 — Additional file 6: Figure S2. Construction of LASSO Cox regression model in TCGA-PRAD. [file 12935_2020_1230_MOESM6_ESM.tif]

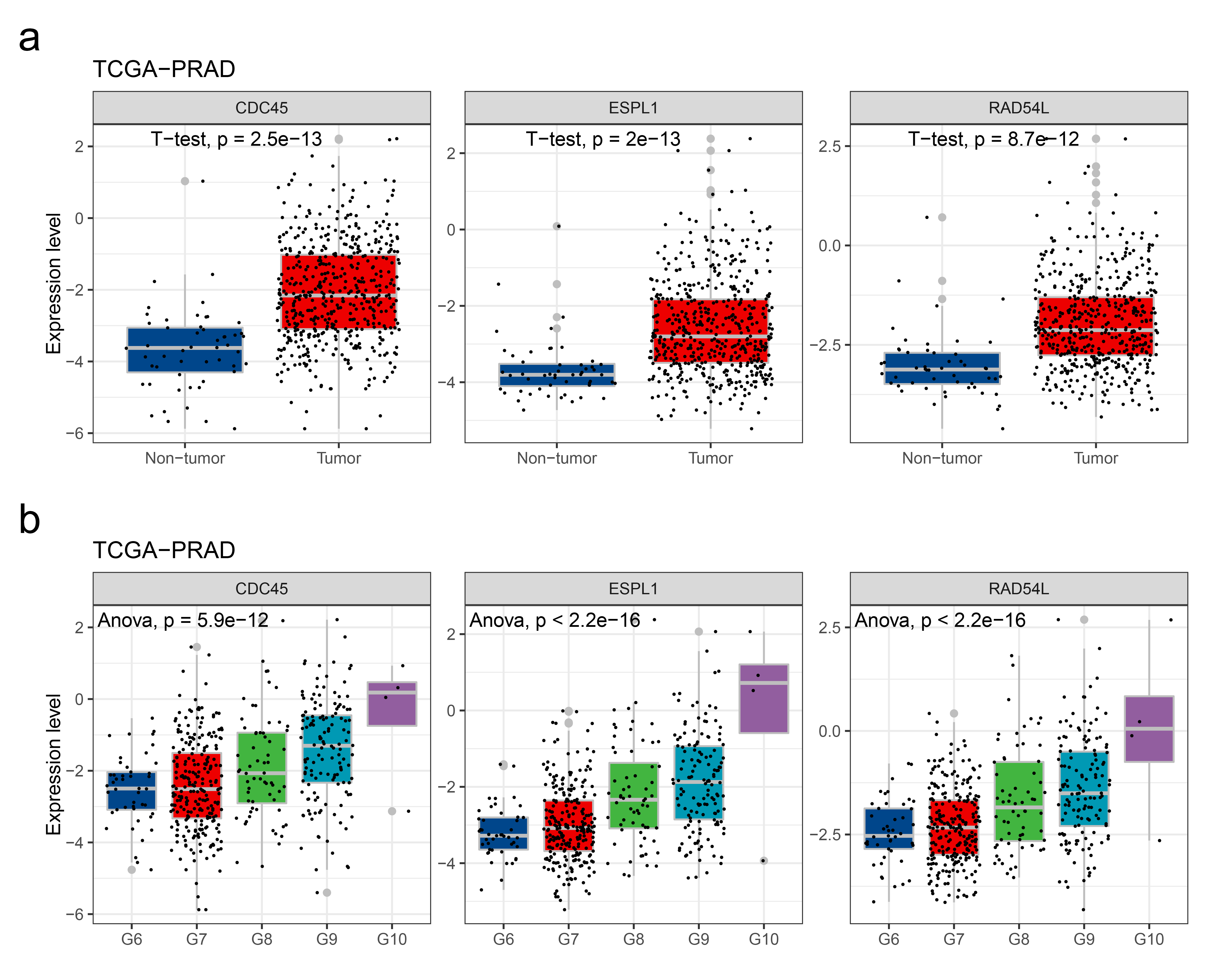

Supplement: Supplementary file 7 — Additional file 7: Figure S3. Expression level of 3 Gleason score related genes. (a) Expression of 3 Gleason score related genes between prostate cancer and normal prostate in TCGA-PRAD. (b) Expression of 3 Gleason score related genes between different Gleason scores in TCGA-PRAD. [file 12935_2020_1230_MOESM7_ESM.tif]

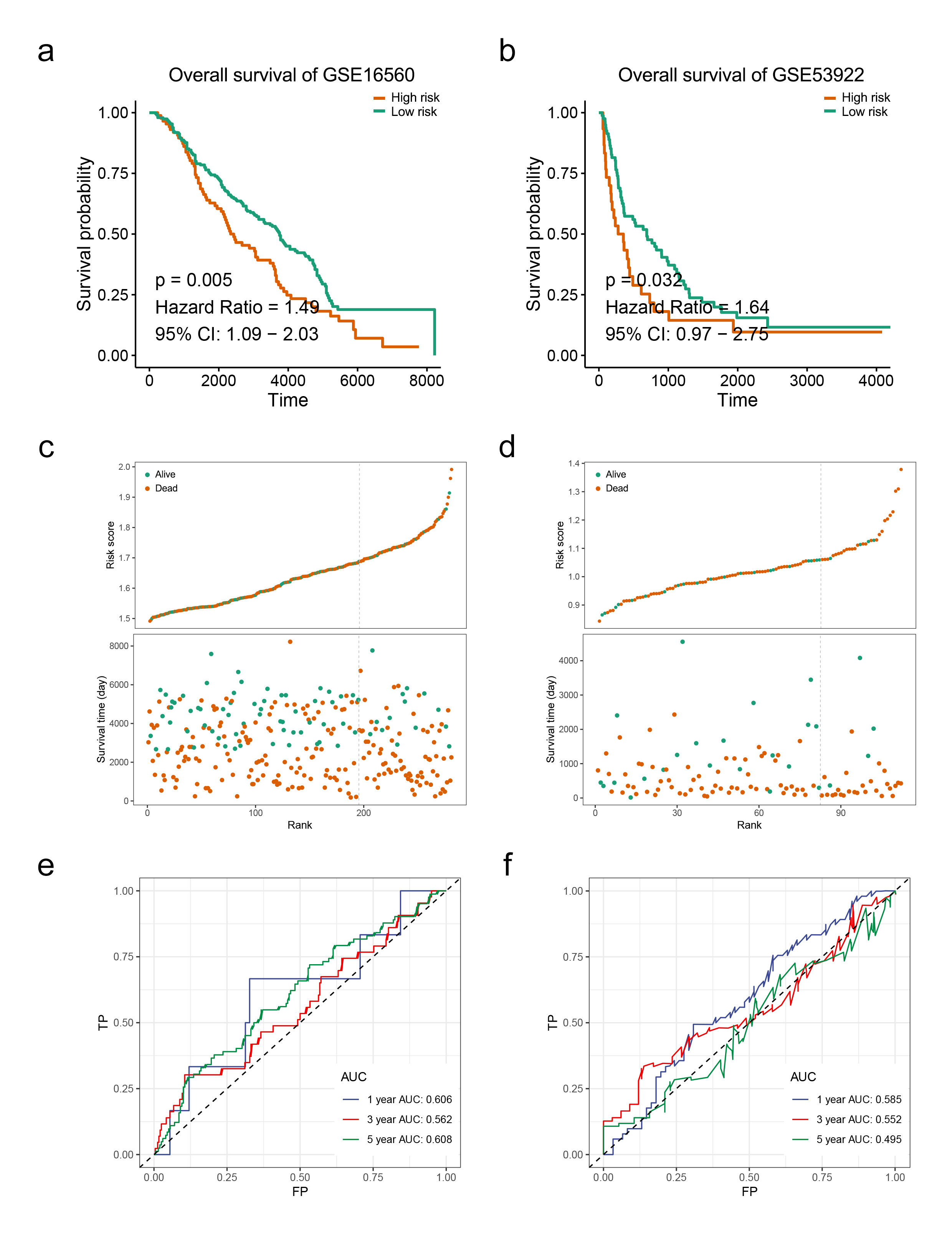

Supplement: Supplementary file 8 — Additional file 8: Figure S4. Risk score derived from 3-gene signature is a prognostic biomarker for overall survival (OS). (a, c, e) KM survival, risk score and time-dependent ROC curves of OS in GSE16560 validation cohort. (b, d, f) KM survival, risk score and time-dependent ROC curves of OS in GSE53922 validation cohort. The high-risk and low-risk groups were stratified at optimal cut-off due to the risk score. The AUC was assessed at 1, 3 and 5 years. [file 12935_2020_1230_MOESM8_ESM.tif]

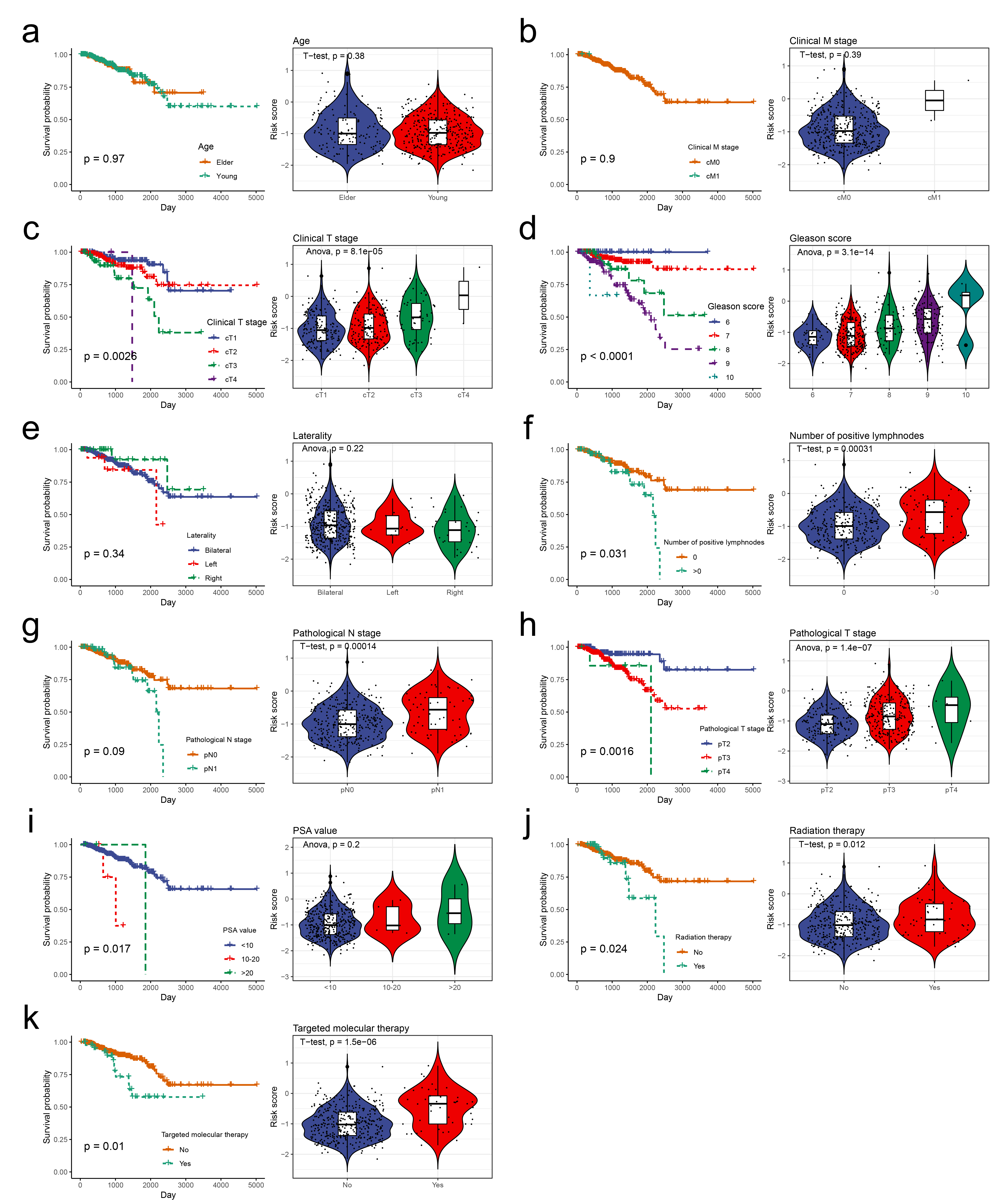

Supplement: Supplementary file 10 — Additional file 10: Figure S5. Association between risk score and clinicopathological characteristics. The survival rate of indicated subgroups in different clinicopathological characteristics was measured. Boxplots indicate the correlation between risk score and the indicated subtype of each clinicopathological characteristics by the t-test or one-way ANOVA. The patients are stratified into different subtypes based on (a) age: elder: age ≥ 65, younger: age < 65. (b) clinical M stage: cM0 and cM1. (c) clinical T stage: cT1, cT2, cT3 and cT4. (d) total Gleason score: 6, 7, 8, 9, 10. (E) laterality: left, right and bilateral. (f) number of positive lymph nodes by HE: number of positive lymph nodes by HE = 0 and number of positive lymphnodes by HE > 0. (g) pathological N stage: pN0 and pN1. (h) pathological N stage: pT2, pT3 and pT4. (i) PSA value: <10, 10-20 and >20. (j) Radiation therapy: yes and no. (k) targeted molecular therapy: yes and no. [file 12935_2020_1230_MOESM10_ESM.tif]

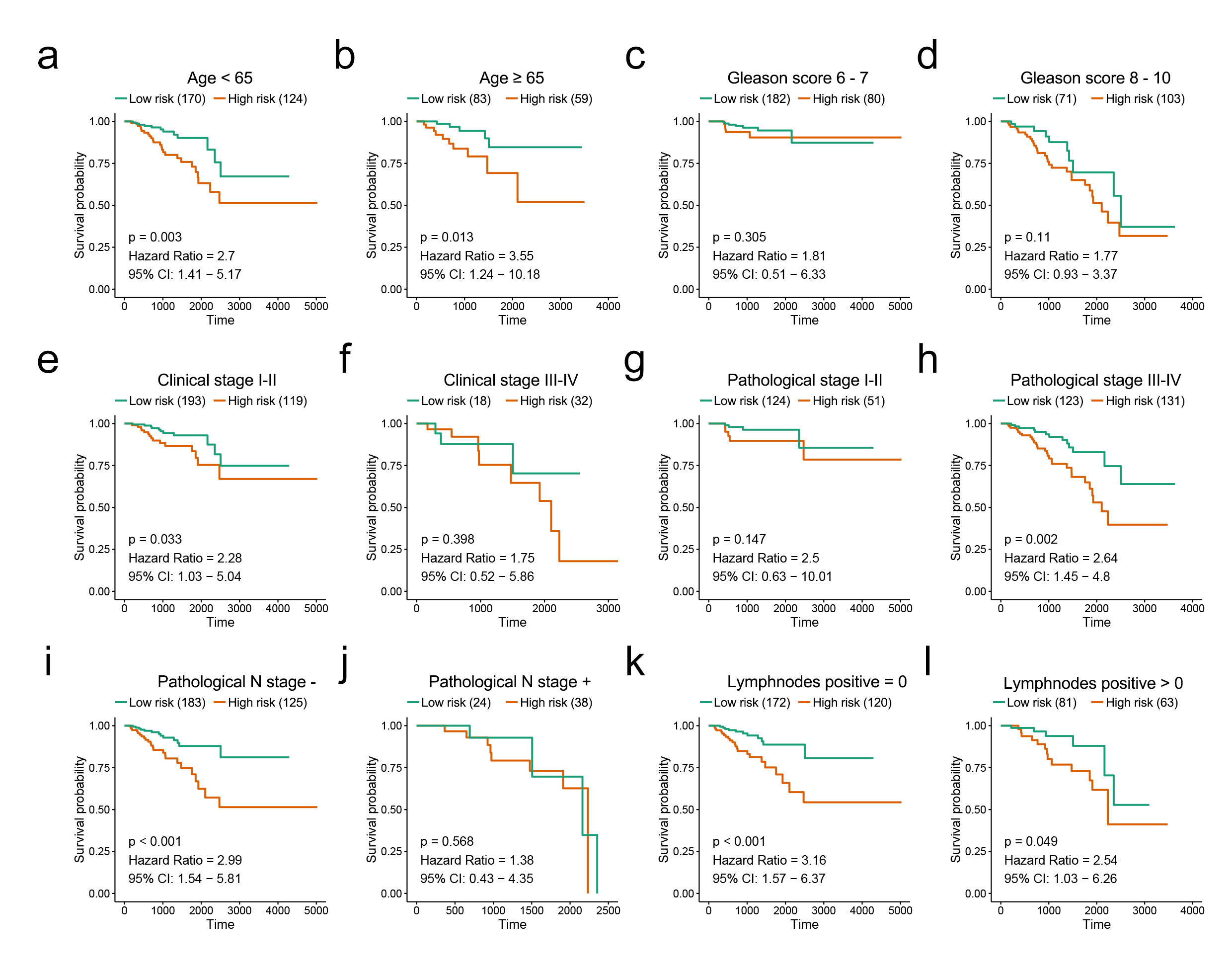

Supplement: Supplementary file 11 — Additional file 11: Figure S6. KM survival subgroup analyses of all patients in TCGA-PRAD cohort according to the risk score stratified by clinical characteristics. (a) Age < 65. (b) Age ≥ 65. (c) Gleason score 6-7. (d) Gleason score 8-10. (e) Clinical stage I-II. (f) Clinical stage III-IV. (g) Pathological stage I-II. (h) Pathological stage III-IV. (i) Pathological N stage - (N0). (j) Pathological N stage + (N+). (k) Number of positive lymph nodes by HE = 0. (l) Number of positive lymph nodes by HE > 0. [file 12935_2020_1230_MOESM11_ESM.tif]

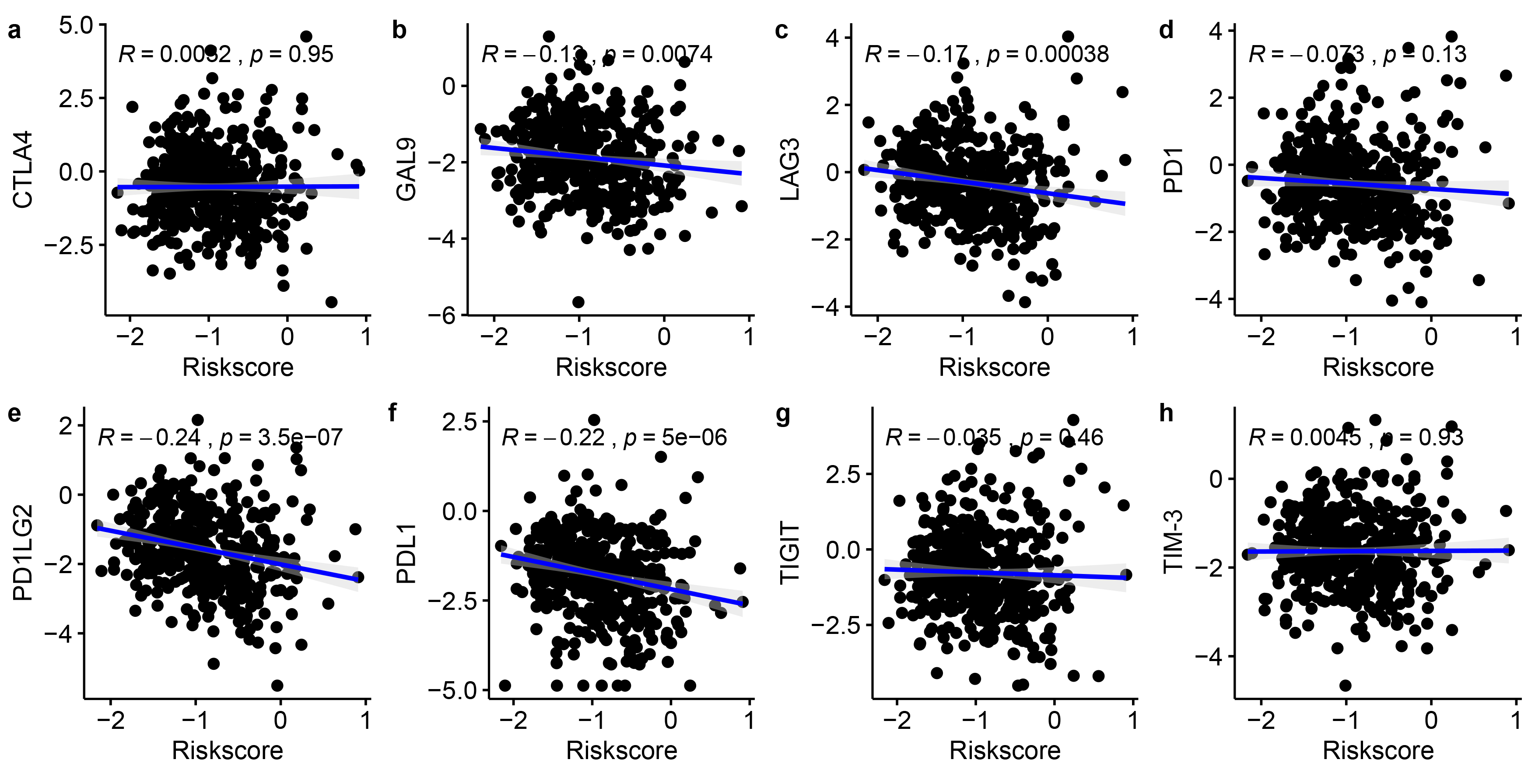

Supplement: Supplementary file 16 — Additional file 16: Figure S7. Correlation between risk score and immune checkpoints. [file 12935_2020_1230_MOESM16_ESM.tif]
