# Supplementary material for: A Gleason score-related outcome model for human prostate cancer: a comprehensive study based on weighted gene co-expression network analysis
Source: Cancer Cell Int. 2020 May 11;20:159. doi: 10.1186/s12935-020-01230-x (PMC7216484; doi:10.1186/s12935-020-01230-x)
Supplement: Supplementary file 9 — Additional file 9: Table S5. Clinical information with DFS data of TCGA-PRAD. [file 12935_2020_1230_MOESM9_ESM.docx]

Clinical information with DFS data of TCGA-PRAD

| Clinical feature | | Patient number |
| --- | --- | --- |
| Age | <65 | 294 |
|  | ≥65 | 142 |
| Laterality | Left | 17 |
|  | Right | 30 |
|  | Bilateral | 383 |
| Clinical T stage | cT1 | 162 |
|  | cT2 | 150 |
|  | cT3 | 48 |
|  | cT4 | 2 |
| Clinical M stage | cM0 | 401 |
|  | cM1 | 2 |
| Pathological T stage | pT2 | 175 |
|  | pT3 | 246 |
|  | pT4 | 8 |
| Pathological N stage | cN0 | 308 |
|  | cN1 | 62 |
| Gleason_score | 6 | 43 |
|  | 7 | 219 |
|  | 8 | 60 |
|  | 9 | 110 |
|  | 10 | 4 |
| PSA_value | <10 | 377 |
|  | 10-20 | 5 |
|  | ≥20 | 3 |
| Number of positive lymphnodes | 0 | 292 |
|  | >0 | 63 |
| Radiation therapy | Yes | 54 |
|  | No | 363 |
| Targeted molecular therapy | Yes | 45 |
|  | No | 371 |
